# 2496: Improving minority health and reducing health disparities: Research at the intersection of health disparity science and clinical and translational science

**DOI:** 10.1017/cts.2017.285

**Published:** 2018-05-10

**Authors:** Meryl Sufian, Derrick Tabor, Phuong-Tu Le

**Affiliations:** National Institutes of Health, Bethesda, MD, USA

## Abstract

OBJECTIVES/SPECIFIC AIMS: (1) To explain and discuss minority health and health disparities and the mechanisms, for example, individual behaviors and lifestyle, genetics and epigenetics, physical and cultural environment, and clinical events and health care, that lead to health disparities. (2) To explore the intersection between health disparity science and clinical and translational science. (3) To present and discuss the NIMHD Framework and how it can be used to guide multilevel research to address minority health and health disparities. (4) To highlight examples of NIMHD-funded novel and innovative research relevant to clinical and translational research from a health disparities perspective. METHODS/STUDY POPULATION: The NIMHD Research Framework will be introduced that is currently being used by NIMHD to address minority health and health disparity research. The Framework looks at targeted populations in relation to biological, behavioral, physical, and sociocultural environmental domains of influence as well as the health care system. These domains have different levels of influence: individual, family/interpersonal, community, and population. Targeted populations include NIH-designated health disparity populations, that include racial/ethnic minorities, socioeconomically disadvantaged populations, underserved rural populations, and sexual and gender minorities. The following research opportunities are among the many opportunities offered by NIMHD: Disparities in Surgical Care and Outcomes Social Epigenomics for Minority Health and Health Disparities Addressing Health Disparities Among Immigrant Populations. RESULTS/ANTICIPATED RESULTS: Select examples of NIMHD supported minority health and health disparities research that intersects with clinical and translational research will be presented. Candidate examples include: Genetic Architecture of Lupus (SLE) in individuals with Asian ancestry; A Novel Racial Disparity Marker for Risk Prediction in Triple Negative Breast Cancer Patients; Self-Applied Wearable Ultrasound Therapy for Osteoarthritis Management in Rural Central NY; Design and Development of a Multifunctional Self-service Health Screening Kiosk. DISCUSSION/SIGNIFICANCE OF IMPACT: Despite notable improvements gained as a result of medical and scientific advances, there continues to be an alarming disproportionate burden of illness and lack of representation in research among minority and other socially disadvantaged and underserved populations. To meet this challenge, NIMHD is committed to supporting a wide range of clinical and translational research aimed at the development of innovative strategies and approaches to reduce and, eventually, eliminate health disparities. NIMHD’s mission, research priorities, and funding opportunities are relevant to the efforts and interests of clinical and translational scientists, especially those interested in the translation of research findings into interventions, products, and tools that may improve minority health and quality of life, increase adherence to medication and treatment regimens, increase access, and improve the delivery of health services.

